# Differing Impact of Preterm Birth on the Right and Left Atria in Adulthood

**DOI:** 10.1161/JAHA.122.027305

**Published:** 2022-12-06

**Authors:** Art Schuermans, Tamara den Harink, Betty Raman, Robert W. Smillie, Maryam Alsharqi, Afifah Mohamed, Winok Lapidaire, Arend W. van Deutekom, Paul Leeson, Adam J. Lewandowski

**Affiliations:** ^1^ Oxford Cardiovascular Clinical Research Facility, Division of Cardiovascular Medicine, Radcliffe Department of Medicine University of Oxford Oxford United Kingdom; ^2^ Department of Cardiovascular Sciences KU Leuven Leuven Belgium; ^3^ Department of Epidemiology and Data Science, Amsterdam UMC University of Amsterdam Amsterdam The Netherlands; ^4^ Oxford Centre for Clinical Magnetic Resonance Research, Division of Cardiovascular Medicine, Radcliffe Department of Medicine University of Oxford Oxford United Kingdom; ^5^ Oxford University Hospitals NHS Foundation Trust Oxford United Kingdom; ^6^ Department of Cardiac Technology College of Applied Medical Sciences, Imam Abdulrahman Bin Faisal University Dammam Saudi Arabia; ^7^ Department of Diagnostic Imaging & Applied Health Sciences, Faculty of Health Sciences Universiti Kebangsaan Malaysia Kuala Lumpur Malaysia; ^8^ Department of Paediatrics, Division of Paediatric Cardiology Erasmus MC‐Sophia Children’s Hospital Rotterdam The Netherlands

**Keywords:** cardiac remodeling, cardiovascular diseases, magnetic resonance imaging, preterm birth, transitional physiology, Developmental biology, Physiology, Cardiovascular Disease, Remodeling, Magnetic Resonance Imaging (MRI)

## Abstract

**Background:**

Preterm birth affects 10% of live births and is associated with an altered left ventricular and right ventricular phenotype and increased cardiovascular disease risk in young adulthood. Because left atrial (LA) and right atrial (RA) volume and function are known independent predictors of cardiovascular outcomes, we investigated whether these were altered in preterm‐born young adults.

**Methods and Results:**

Preterm‐born (n=200) and term‐born (n=266) adults aged 18 to 39 years underwent cardiovascular magnetic resonance imaging. LA and RA maximal and minimal volumes (absolute, indexed to body surface area, and as a ratio to ventricular volumes) were obtained to study atrial morphology, while LA and RA stroke volume, strain, and strain rate were used to assess atrial function. Secondary analyses consisted of between‐group comparisons based on degree of prematurity. Absolute RA volumes and RA volumes indexed to right ventricular volumes were significantly smaller in preterm‐born compared with term‐born adults. In addition, RA reservoir and booster strain were higher in preterm‐born adults, possibly indicating functional compensation for the smaller RA volumes. LA volumes indexed to left ventricular volumes were significantly greater in preterm‐born adults as compared with term‐born adults, although absolute LA volumes were similar between groups. LA and RA changes were observed across gestational ages in the preterm group but were greatest in those born very‐to‐extremely preterm.

**Conclusions:**

Preterm‐born adults show changes in LA and RA structure and function, which may indicate subclinical cardiovascular disease. Further research into underlying mechanisms, opportunities for interventions, and their prognostic value is warranted.

**Nonstandard Abbreviations and Acronyms**
B: unstandardized regression coefficient
LA: left atrial/left atrium
RA: right atrial/right atrium

**Clinical Perspective**

**What Is New?**

- Atrial size and function are known predictors of cardiovascular outcomes but have not yet been comprehensively investigated in preterm‐born adults, a high‐risk group for cardiovascular diseases.
- In this study, cardiovascular magnetic resonance profiling in a large cohort of young adults born preterm showed that preterm birth is associated with distinct changes in left and right atrial structure and function suggestive of subclinical disease.

**What Are the Clinical Implications?**

- These findings may be relevant for acquiring a better understanding of the long‐term cardiovascular implications of being born preterm and building toward clinical monitoring and preventive strategies.
- Screening for left atrial and right atrial irregularities and measurements in preterm‐born individuals could ultimately become a valuable component of an integrated screening approach to aid in modifying the lifetime cardiovascular risk of people born preterm.

Globally, >10% of live births are preterm (<37 weeks' gestation).^1^ Preterm‐born adults are at an increased risk for early heart failure,^2^, ^3^ ischemic heart disease,^4^ and cardiovascular mortality,^5^ possibly in part because of alterations in left ventricular (LV) and right ventricular (RV) structure and function.^6^, ^7^ Many studies have identified the importance of the ventricles in the pathophysiology of cardiovascular diseases.^8^, ^9^ Although the atria have also been recognized to play a significant and independent role in cardiovascular disease,^10^, ^11^, ^12^, ^13^, ^14^, ^15^ to our knowledge, no study has specifically focused on the long‐term effects of prematurity on atrial remodeling.

The atria receive, store, and transport blood into the ventricles. These functions are conventionally divided into 3 phases: (1) a reservoir phase, in which the atria store venous return; (2) a conduit phase, in which the atria passively transfer blood to the ventricles; and (3) a booster phase, in which ventricular filling is augmented by atrial contraction.^16^, ^17^ The atria are susceptible to volume and pressure overload and can remodel to compensate for ventricular impairments.^18^, ^19^ Because the ventricles of preterm‐born adults are physiologically altered in comparison to those in adults born at term,^20^, ^21^, ^22^ the atria may differ proportionally or display greater remodeling to adapt to ventricular functional deficits.

Cardiovascular magnetic resonance (CMR) imaging is currently the gold standard for noninvasive estimations of cardiac dimensions, including left atrial (LA) and right atrial (RA) volumes. In addition, 2‐dimensional CMR tissue tracking allows for the evaluation of myocardial deformation, including atrial strain and strain rate,^23^, ^24^ and has become a robust tool as part of clinical routine for evaluating myocardial performance.^25^, ^26^ We have therefore performed the first CMR imaging study in a large, prospective cohort of young adults to investigate whether preterm birth is associated with differences in LA and RA structure and function compared with individuals born at term. Given the extent of the RV and LV changes observed in this population, we hypothesized that LA and RA structure and function would also be adversely affected in preterm‐born young adults compared with their term‐born peers.

## METHODS

The data that support the findings of this study are available from the corresponding author upon reasonable request.

### Study Population

We assessed individuals aged 18 to 39 years cross‐sectionally as part of an ongoing program of research into the impact of preterm birth on cardiac remodeling in Oxford, United Kingdom.^22^ All participants had been identified through open recruitment in the local community using posters and e‐mails, mailed invitations from the John Radcliffe Hospital birth registries, patient invitation through the local hypertension clinic or via general practice records, as well as prospective follow‐up from birth. Participants with a body mass index (BMI) >40 kg/m^2^, history of heart disease, cerebrovascular disease, hypertension treatment, or missing/incomplete CMR files were excluded. All research visits took place at the Oxford Cardiovascular Clinical Research Facility and the Oxford Centre for Clinical Magnetic Resonance Research, where participants underwent anthropometric, demographic, and cardiovascular phenotyping. To ensure anonymity and blinded analysis, data were coded with subject‐ and study‐specific identifications. The South Central Oxford A research ethics committee (06/Q1604/118), the South Central Berkshire research ethics committee (14/SC/0275), and the South Central Oxford B research ethics committee (16/SC/0016) granted ethical approval. All participants provided signed informed consent.

### Study Visit

#### Anthropometry, Blood Samples, and Blood Pressure Measurements

All subjects attended the research unit after a fast of at least 4 hours. Height, weight, and blood pressure measurements were collected by trained clinical research professionals, as described previously.^20^, ^22^, ^27^, ^28^ Venous blood samples were drawn at rest and subsequently centrifuged, separated within 30 minutes, and stored at −80 °C. Data on birth characteristics, medical history, smoking behavior, and family history were acquired via prospective collection, access to medical notes, or self‐reported questionnaire.

#### Cardiac Magnetic Resonance Imaging

CMR was performed on a 1.5‐Tesla or 3.0‐Tesla magnetic resonance scanner (1.5‐Tesla Sonata and 3‐Tesla TIM Trio; Siemens Medical Solutions). The feasibility and consistency of cardiac volume, mass, and strain measurements across field strengths were demonstrated in earlier studies.^29^, ^30^ Horizontal and vertical long‐axis electrocardiographically gated steady‐state free precession cine images were obtained, followed by short‐axis steady‐state free precession cine images.^20^ Data were acquired at end‐expiratory breath‐hold and digitally stored for postprocessing.

#### Image Analysis

All CMR analysis was carried out using analytical software (cvi42, Circle Cardiovascular Imaging Inc, Calgary, Alberta, Canada).

#### Quantification of Ventricular Volumes, Mass, and Function

To evaluate LV and RV volumes and mass, both the epicardial and endocardial borders were manually contoured on short‐axis cine images for each slice at end‐diastole and endocardial borders at end‐systole.^22^ The end‐diastolic and end‐systolic cardiac phases, as well as basal and apical LV and RV slices, were visually determined as previously described.^20^, ^21^ All volumes and masses were reported unindexed and indexed to body surface area (BSA).

#### Quantification of Atrial Volumes and Function

**Figure 1:**
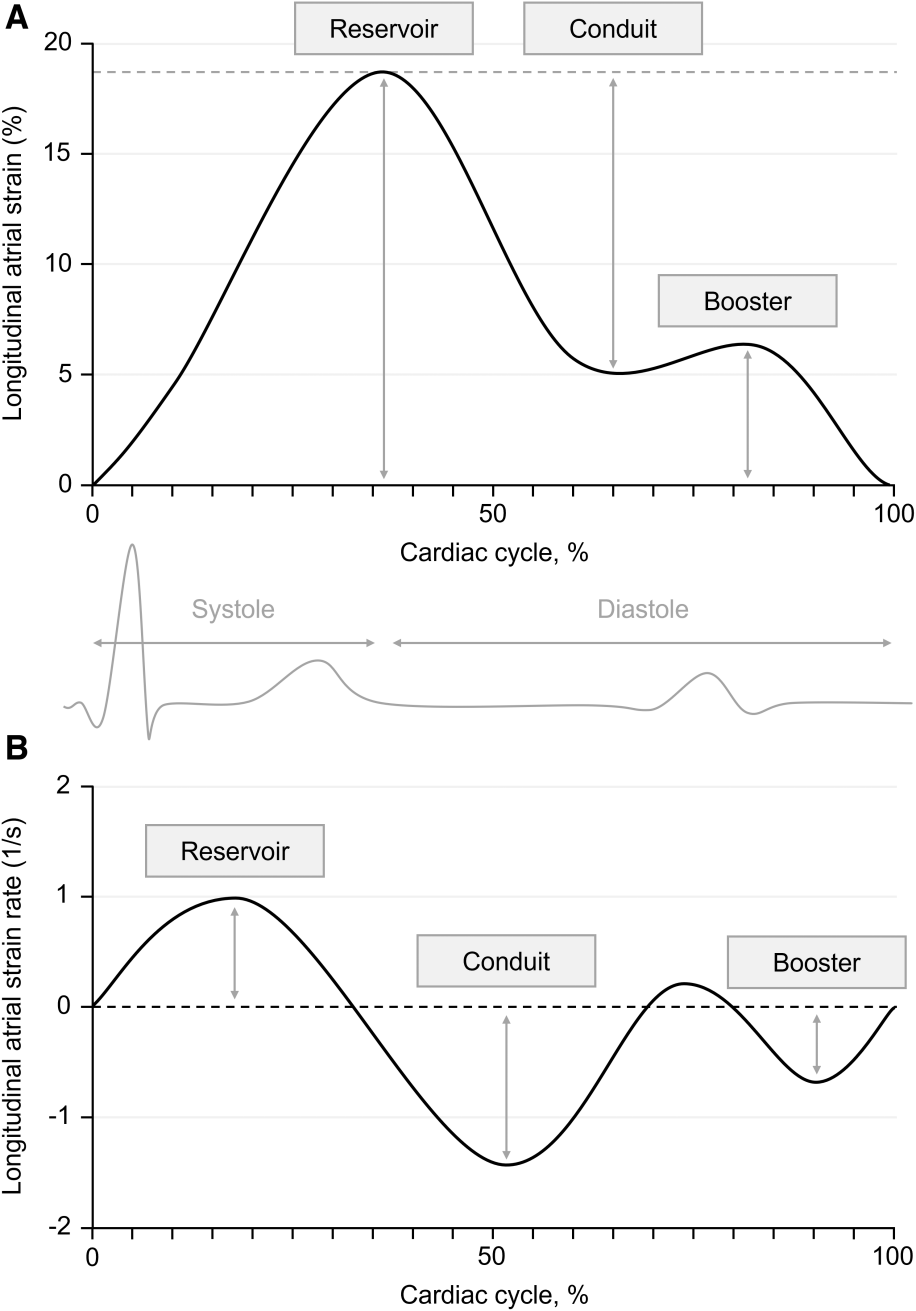
Atrial strain and strain rate. **A**, Graph demonstrating the 3 components of atrial strain (ie, reservoir strain, conduit strain, and booster strain) during the cardiac cycle. **B**, Graph demonstrating the 3 components of atrial strain rate (ie, reservoir strain rate, conduit strain rate, and booster strain rate) during the cardiac cycle.

To evaluate minimal and maximal atrial volumes, the endocardial borders were manually contoured on long‐axis cine images at ventricular end‐diastole and end‐systole, respectively. Neither the atrial appendages, pulmonary veins, or venae cavae were included in the contours. LA and RA peak longitudinal reservoir, conduit and booster strain, and strain rate were assessed using an automated tracking algorithm.^23^, ^31^ Strain and strain rate components (Figure 1) were derived from cine images by 2‐dimensional tissue tracking analysis using previously reported methodology.^23^ All LA values were based on composite estimates from the vertical and horizontal long‐axis views, while RA values were based on horizontal long‐axis views only. For each participant, LA and RA stroke volumes were defined as the difference between maximal and minimal volumes, and ejection fractions as stroke volumes divided by maximal volumes. All volumes were reported unindexed and indexed to BSA. To assess atrial volumes relative to ventricular volumes, ratios between atrial and corresponding ventricular volumes were calculated for each participant (ie, atrial maximal volume to ventricular end‐diastolic volume, atrial minimal volume to ventricular end‐systolic volume, and atrial stroke volume to ventricular stroke volume). Additionally, ratios between atrial and ventricular volumes at end‐systolic time point (ie, atrial maximal volume to ventricular end‐systolic volume) and at end‐diastolic time point (ie, atrial minimal volume to ventricular end‐diastolic volume) were calculated.^32^, ^33^, ^34^

### Statistical Analysis

Primary analyses consisted of direct group comparisons between preterm‐born (<37 weeks' gestation) and term‐born (≥37 weeks' gestation) adults. Secondary analyses consisted of (1) direct group comparisons between preterm‐born and term‐born adults stratified by sex; (2) multivariable linear regression analyses of LA and RA parameters versus gestational age and birth weight; (3) multivariable linear regression analyses of LA and RA parameters versus LV and RV parameters in preterm‐born and term‐born adults, respectively; (4) multivariable linear regression analyses of LA and RA parameters versus cardiovascular risk factors (ie, BMI, mean arterial blood pressure, smoking status) in preterm‐born and term‐born adults, respectively; and (5) direct group comparisons between extremely‐to‐very preterm‐born (<32 weeks' gestation), moderately‐to‐late preterm‐born (≥32 to <37 weeks' gestation), and term‐born adults.

Data were summarized as mean±SD for numerical variables or number (proportion [%]) for categorical variables. *P* values for between‐group comparisons were adjusted for sex and age using multivariable linear regression. In addition, unstandardized coefficients (B) were reported with 95% CI and *P* values. Diagnostic tests for regression models were performed to test for the assumptions of linearity, normal distribution of the residuals, and constant variance. A *P* value <0.05 was considered statistically significant unless multiple subgroup comparisons were made, in which case a *P* value <0.01 was considered statistically significant. All tests were 2‐sided.

To qualitatively compare the shape of the LA and RA strain curves between preterm‐born and term‐born adults, pooled strain curves were constructed for the 2 groups separately using local regression analysis. The intraobserver (A.S.) and interobserver (A.S. and T.d.H.) variability of LA and RA measurements was determined by coefficient of variation for 10 randomly selected participants. All statistical analyses were carried out with SPSS, version 28 (IBM), and R, version 4.0.3 (R Foundation for Statistical Computing).

## RESULTS

### Participant Inclusion and Baseline Characteristics

**Figure 2:**
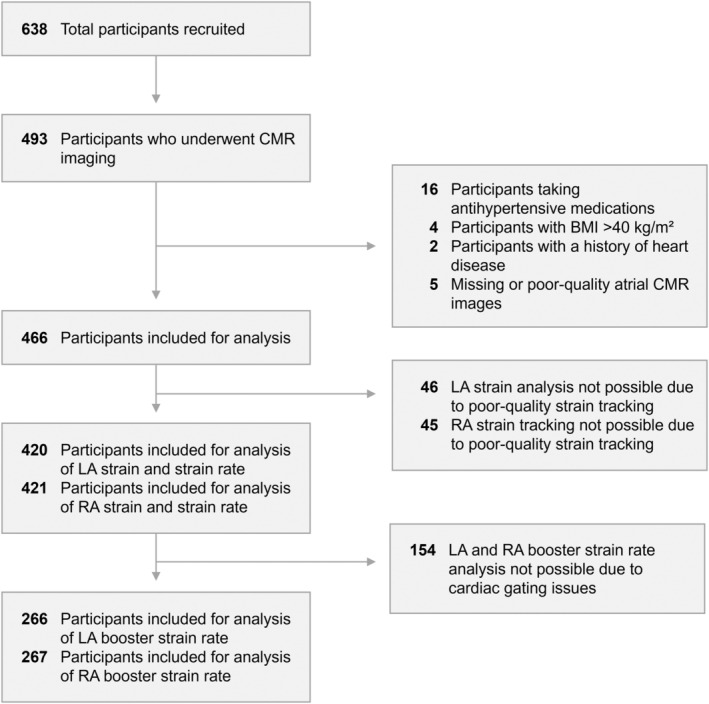
Study flowchart. BMI indicates body mass index; CMR, cardiovascular magnetic resonance; LA, left atrial; and RA, right atrial.

Out of the 638 participants recruited to the Oxford research program on cardiovascular outcomes of preterm birth, 493 underwent CMR imaging (Figure 2). Participants receiving pharmacological treatment for hypertension (n=16), with BMI >40 kg/m^2^ (n=4), or a history of cardiac disease (n=2) were excluded from the present study because of their possible confounding effects on atrial remodeling. In addition, data from 5 participants were excluded because of low image quality. Assessment of LA strain and strain rate was undertaken in 420 out of 466 participants, while RA strain and strain rate were evaluated in 421 participants. LA and RA booster strain rates could only be analyzed in 266 and 267 participants, respectively, because of ≈50% of CMR scans using prospective cardiac gating, which led to the cardiac cycle being terminated during or just after the booster plateau phase.

Of the 466 participants included in the present study, 200 (42.9%) were born preterm (31.4±3.0 weeks' gestation), and 266 (57.1%) were born at term (39.6±1.2 weeks' gestation, Table 1). The cohort was primarily White (>95%). Preterm‐born adults were smaller, weighed less, and had smaller BSA estimates than their term‐born counterparts. Although there were no significant differences in BMI or smoking status between groups, total and low‐density lipoprotein cholesterol, triglyceride, and glucose levels were increased in the preterm cohort. Additionally, resting systolic blood pressure, mean arterial pressure, and heart rate were higher in preterm‐born adults compared with controls.

**Table 1:** Cohort Characteristics

Characteristics	Preterm‐born adults (n=200)	Term‐born adults (n=266)	*P* value
Demographics and anthropometrics			
Age, y	25.7±3.9	26.5±4.6	0.069
Male, n (%)	91 (45.5)	136 (51.1)	0.274
Height, cm	169.6±10.1	173.6±9.2	<0.001
Weight, kg	69.1±13.3	72.4±12.9	0.029
BMI, kg/m^2^	23.9±3.7	23.9±3.4	0.560
BSA, m^2^	1.79±0.20	1.86±0.19	<0.001
Birth weight, g	1635±638	3449±428	<0.001
Birth weight, *z* score	−0.26±1.05	0.09±0.90	<0.001
Small for gestational age, n (%)	10 (5.0)	5 (1.9)	0.054
Gestational age, wk	31.4±3.0	39.6±1.2	<0.001
<28 wk, n (%)	21 (10.5)	…	…
28–31 wk, n (%)	74 (37.0)	…	…
32–36 wk, n (%)	105 (52.5)	…	…
Smoker, n (%)	20 (10.0)	23 (8.6)	0.613
Biochemistry			
Total cholesterol, mmol/L	4.66±1.04	4.44±0.94	0.011
HDL‐C, mmol/L	1.49±0.37	1.43±0.35	0.243
LDL‐C, mmol/L	2.78±0.79	2.58±0.78	0.002
Triglycerides, mmol/L	1.23±0.97	0.98±0.57	<0.001
High‐sensitivity CRP, mg/L	1.92±3.55	1.56±3.16	0.262
Glucose, mmol/L	4.96±0.43	4.81±0.47	<0.001
Insulin, pmol/L	53.48±30.81	47.34±45.31	0.075
Brachial blood pressure and heart rate			
Resting systolic blood pressure, mm Hg	120.9±11.5	118.0±11.2	<0.001
Resting diastolic blood pressure, mm Hg	72.9±8.1	71.8±8.7	0.064
Resting mean arterial pressure, mm Hg	88.9±8.4	87.2±8.7	0.007
Resting heart rate, bpm	71.2±10.5	66.1±9.7	<0.001

Group characteristics presented as mean±SD or n (%). *P* values represent between‐group comparisons that were adjusted for differing sex and age distributions using multivariable linear regression. BMI indicates body mass index; bpm, beats per minute; BSA, body surface area; CRP, C‐reactive protein; HDL‐C, high‐density lipoprotein cholesterol; and LDL‐C, low‐density lipoprotein cholesterol.

### Reproducibility of LA and RA Measurements

Our group and others have previously established the reproducibility of LV and RV volume and mass measurements using CMR imaging.^21^, ^35^, ^36^ For all LA and RA volumetric, strain, and strain rate measurements, intraobserver and interobserver coefficients of variation were similar to those reported in previous studies (Table S1).^23^, ^24^, ^37^, ^38^

### Potentially Greater Impact of Preterm Birth on the LV Than LA in Young Adulthood

As previously reported,^22^ preterm‐born adults showed smaller LV volumes, including end‐diastolic, end‐systolic, and stroke volume indexed to BSA, while LV myocardial mass index was increased (Table 2). Furthermore, LV ejection fraction was significantly reduced in the preterm‐born cohort, as was LV cardiac index.

**Table 2:** Left Ventricular and Left Atrial Structure and Function in Preterm‐Born and Term‐Born Adults

	Preterm‐born adults (n=200)	Term‐born adults (n=266)	*P* value
LV structure and function			
End‐diastolic volume index, mL/m^2^	72.7±10.6	81.0±12.6	<0.001
End‐systolic volume index, mL/m^2^	27.0±5.8	28.8±7.1	0.002
Stroke volume index, mL/m^2^	45.8±7.6	52.2±8.2	<0.001
Myocardial mass index, g/m^2^	64.4±10.3	55.6±9.7	<0.001
Ejection fraction, %	63.1±5.6	64.7±5.2	0.002
Cardiac index, L/min per m^2^	3.2±0.6	3.4±0.7	0.003
LA structure and function			
Max. volume, mL	58.4±16.2	61.9±17.6	0.073
Min. volume, mL	21.0±7.8	22.3±8.8	0.231
Stroke volume, mL	37.4±10.2	39.6±10.6	0.054
Max. volume index, mL/m^2^	32.5±7.6	33.3±8.6	0.386
Min. volume index, mL/m^2^	11.7±3.8	11.9±4.4	0.614
Stroke volume index, mL/m^2^	20.8±4.9	21.4±5.3	0.326
Ejection fraction, %	64.5±6.6	64.8±7.2	0.552
Reservoir strain, %	20.0±3.0	20.1±3.2	0.346
Conduit strain, %	15.4±2.8	15.7±3.0	0.111
Booster strain, %	5.9±2.1	5.8±1.9	0.513
Reservoir strain rate, 1/s	0.90±0.16	0.92±0.19	0.054
Conduit strain rate, 1/s	−1.54±0.35	−1.50±0.72	0.649
Booster strain rate, 1/s	−0.76±0.27	−0.67±0.27	0.016
LA volumes indexed to LV volumes			
LA max. volume to LV end‐diastolic volume ratio	0.45±0.09	0.41±0.10	<0.001
LA min. volume to LV end‐systolic volume ratio	0.44±0.15	0.43±0.17	0.315
LA stroke volume to LV stroke volume ratio	0.46±0.10	0.41±0.09	<0.001
LA max. volume to LV end‐systolic volume ratio	1.24±0.34	1.21±0.38	0.252
LA min. volume to LV end‐diastolic volume ratio	0.16±0.05	0.15±0.05	0.004

Group characteristics presented as mean±SD. *P* values represent between‐group comparisons that were adjusted for differing sex and age distributions using multivariable linear regression. LA indicates left atrial; and LV, left ventricular.

**Figure 3:**
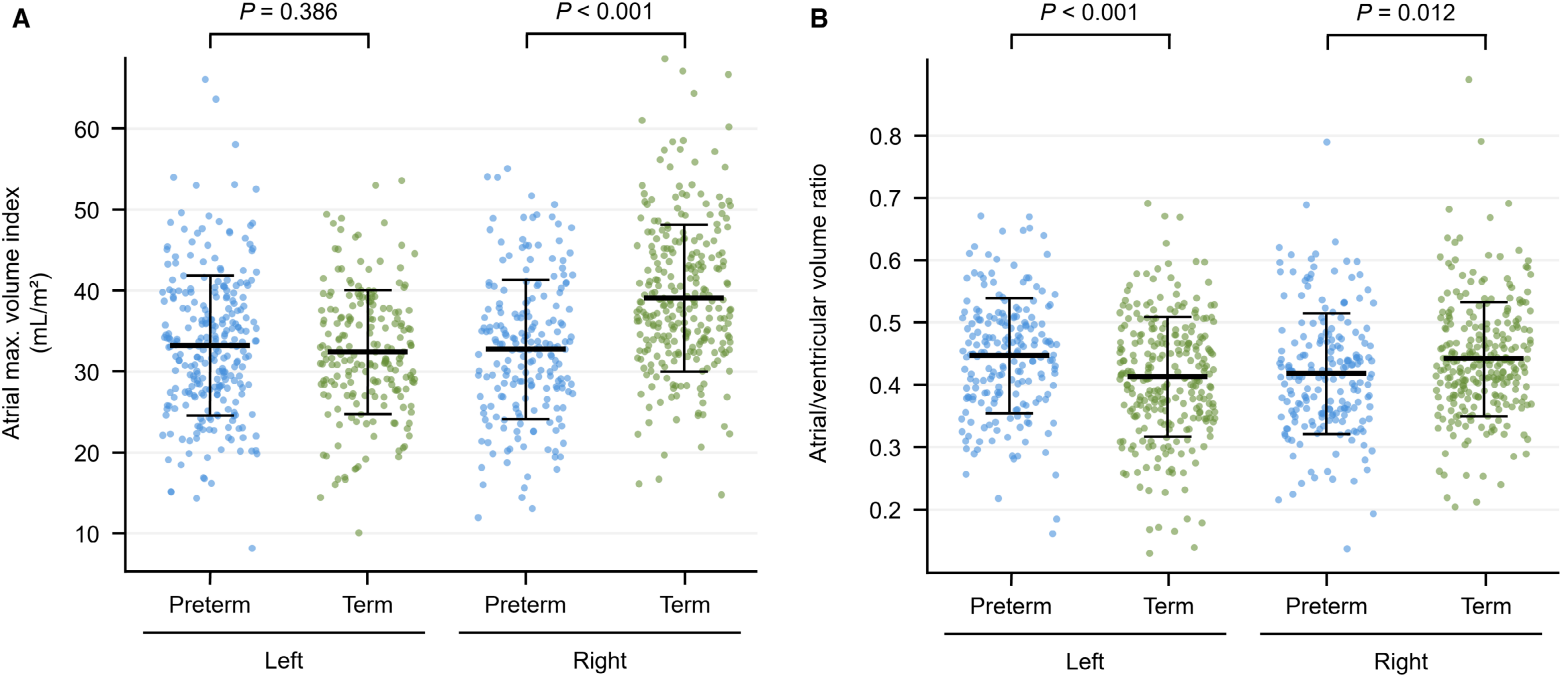
Differences in left and right atrial structure between preterm‐born and term‐born adults. **A**, Preterm‐born adults (blue) showed similar LA maximal volume indexes but smaller RA maximal volume indexes compared with term‐born adults (green). **B**, Preterm‐born adults showed greater LA/LV volume ratios (ie, LA maximal volume to LV end‐diastolic volume ratios) but smaller RA/RV volume ratios (ie, RA maximal volume to RV end‐diastolic volume ratios). Box‐and‐whisker plots presented as mean and SD. *P* values represent between‐group comparisons that were adjusted for differing sex and age distributions using multivariable linear regression. LA indicates left atrial; LV, left ventricular; RA, right atrial; and RV, right ventricular.

LA volumes did not significantly differ between groups. Indeed, absolute and indexed LA maximal, minimal, and stroke volumes were similar between preterm‐born and term‐born adults (Table 2, Figure 3A). Similarly, there were no differences in LA ejection fraction between groups. In addition, LA reservoir, conduit, and booster strain values were similar in preterm‐born compared with term‐born adults (Figure S1A), as were the pooled LA strain curves (Figure S2A). While LA reservoir and conduit strain rates did not significantly differ between groups, LA booster strain rate was greater in preterm‐born than term‐born adults (−0.76±0.27/s versus −0.67±0.27/s, *P*=0.016).

The ratio between LA maximal volume and LV end‐diastolic volume was higher in preterm‐born compared with term‐born adults (0.45±0.10 versus 0.41±0.10, *P*<0.001, Table 2, Figure 3B). Although the ratio between LA minimal volume and LV end‐systolic volume did not significantly differ between groups, the mean LA stroke volume to LV stroke volume ratio was higher in the preterm‐born cohort (0.46±0.10 versus 0.41±0.09, *P*<0.001). Additionally, between‐group comparisons of the LA‐to‐LV volume ratios at end‐systole (ie, LA maximal volume to LV end‐systolic volume) and end‐diastole (ie, LA minimal volume to LV end‐diastolic volume) were made. These parameters are emerging as important measures of atrioventricular coupling and may indicate elevated LV filling pressures.^32^, ^33^, ^34^ We found that the LA‐to‐LV volume ratio at the end‐systolic time point was similar in both groups, while the LA‐to‐LV volume ratio at the end‐diastolic time point was significantly higher in preterm‐born adults (0.16±0.05 versus 0.15±0.05, *P*=0.004).

The between‐group differences in LA booster strain rate and LA‐to‐LV volume ratios were similar among male and female participants (Table S2). Multivariable linear regression analysis showed that these parameters were associated with gestational age rather than birth weight *z* score (Table S3). There was a significant inverse association between LA booster strain rate and BMI in the preterm‐born cohort (*B* [95% CI]: −0.017/s [−0.033; −0.001] per 1‐kg/m^2^ elevation in BMI, *P*=0.037; Table S4), which was not significant in the term‐born cohort (*B* [95% CI]: −0.007/s [−0.020; 0.007] per 1‐kg/m^2^ elevation in BMI, *P*=0.337). Additionally, smoking was associated with lower LA‐to‐LV stroke volume ratios in the preterm group (*B* [95% CI]: −0.071 [−0.115; −0.026] if a current smoker, *P*=0.002), whereas it was associated with higher LA‐to‐LV ratios in the term group (*B* [95% CI]: 0.062 [0.023; 0.101] if a current smoker, *P*=0.002). LA‐to‐LV ratios were inversely associated with LV end‐diastolic volume index in both the preterm and term groups (Table S5). In addition, the LA‐to‐LV stroke volume ratio was significantly and inversely associated with LV ejection fraction in the preterm‐born adults (*B* [95% CI]: −0.003 [−0.006; −0.001] per 1% elevation in LV ejection fraction, *P*=0.007), while this association was not statistically significant in the term‐born adults (*B* [95% CI]: −0.001 [−0.003; 0.001] per 1% elevation in LV ejection fraction, *P*=0.382).

### Smaller RV and RA Volumes and Altered RA Function in Young Adults Born Preterm

Preterm‐born adults had smaller RV end‐diastolic, end‐systolic, and stroke volume indexes than term‐born adults (Table 3). Furthermore, they had a higher RV myocardial mass index, lower RV ejection fraction, and reduced RV cardiac index.

**Table 3:** Right Ventricular and Right Atrial Structure and Function in Preterm‐Born and Term‐Born Adults

	Preterm‐born adults (n=200)	Term‐born adults (n=266)	*P* value
RV structure and function			
End‐diastolic volume index, mL/m^2^	78.8±13.4	89.2±14.6	<0.001
End‐systolic volume index, mL/m^2^	34.3±7.8	36.3±8.9	0.011
Stroke volume index, mL/m^2^	44.5±9.7	52.9±8.2	<0.001
Myocardial mass index, g/m^2^	22.4±3.8	19.4±3.1	<0.001
Ejection fraction, %	56.4±7.1	59.6±5.5	<0.001
Cardiac index, L/min per m^2^	3.2±0.8	3.6±2.2	0.004
RA structure and function			
Max. volume, mL	59.4±18.9	73.2±20.1	<0.001
Min. volume, mL	30.1±11.4	38.3±13.1	<0.001
Stroke volume, mL	29.3±11.1	34.9±10.9	<0.001
Max. volume index, mL/m^2^	32.8±8.6	39.2±9.1	<0.001
Min. volume index, mL/m^2^	16.6±5.4	20.4±6.1	<0.001
Stroke volume index, mL/m^2^	16.2±5.5	18.7±5.3	<0.001
Ejection fraction, %	49.3±10.5	48.0±8.8	0.224
Reservoir strain, %	19.5±4.4	18.3±4.1	0.008
Conduit strain, %	14.5±4.2	13.9±3.7	0.259
Booster strain, %	5.8±2.6	5.2±2.6	0.011
Reservoir strain rate, 1/s	1.00±0.27	0.93±0.22	0.005
Conduit strain rate, 1/s	−1.20±0.40	−1.11±0.34	0.027
Booster strain rate, 1/s	−0.73±0.28	−0.66±0.27	0.046
RA volumes indexed to RV volumes			
RA max. volume to RV end‐diastolic volume ratio	0.42±0.10	0.44±0.09	0.012
RA min. volume to RV end‐systolic volume ratio	0.50±0.17	0.58±0.18	<0.001
RA stroke volume to RV stroke volume ratio	0.37±0.14	0.36±0.11	0.190
RA max. volume to RV end‐systolic volume ratio	0.99±0.28	1.12±0.31	<0.001
RA min. volume to RV end‐diastolic volume ratio	0.12±0.03	0.12±0.03	0.033

Group characteristics presented as mean±SD. *P* values represent between‐group comparisons that were adjusted for differing sex and age distributions using multivariable linear regression. RA indicates right atrial; and RV, right ventricular.

RA maximal volume (59.4±18.9 versus 73.2±20.1 mL, Table 3), minimal volume (30.1±11.4 versus 38.3±13.1 mL), and stroke volume (29.3±11.1 versus 34.9±10.9 mL) were smaller in preterm‐born adults (all *P*<0.001). These differences persisted when indexed to BSA (Figure 3A). Even though RA ejection fraction was similar between preterm‐born and term‐born adults, RA phasic function was higher in preterm‐born adults, because all measures of strain and strain rate (except conduit strain) were greater in the preterm‐born group (Figure S1B). In addition, the pooled RA strain curve had a greater overall amplitude in the preterm than the term group (Figure S2B).

RA maximal and minimal volumes were smaller in preterm‐born adults compared with controls when indexed to RV end‐diastolic (0.42±0.10 versus 0.44±0.09, *P*=0.012, Table 3, Figure 3B) and end‐systolic (0.50±0.17 versus 0.58±0.18, *P*<0.001) volumes. Similarly, the RA‐to‐RV ratios at end‐systolic (0.99±0.28 versus 1.12±0.31, *P*<0.001) and end‐diastolic (0.12±0.03 versus 0.12±0.03, *P*=0.033) time points were increased for the preterm‐born cohort. The RA stroke volume to RV stroke volume ratios were similar between groups.

The between‐group differences in RA structure and function were similar among male and female participants (Table S2). All measures of RA structure and function (except RA‐to‐RV volume ratio at end‐diastolic time point) were significantly associated with gestational age in multivariate regression analyses, whereas none were associated with birth weight *z* score (Table S3). RA stroke volume index was positively associated with BMI in the preterm‐born (*B* [95% CI]: 0.265 mL/m^2^ [0.052; 0.479] per 1‐kg/m^2^ elevation in BMI, *P*=0.016; Table S4) but not the term‐born cohort (*B* [95% CI]: −0.015 mL/m^2^ per 1‐kg/m^2^ elevation in BMI [−0.219; 0.189], *P*=0.884). In addition, RA booster strain was significantly associated with resting mean arterial blood pressure in the term‐born group (*B* [95% CI]: 0.040% [0.002; 0.077] per 1‐mm Hg elevation in resting mean arterial blood pressure, *P*=0.038), but this association did not persist in the preterm‐born group (*B* [95% CI]: 0.006% [−0.042; 0.054] per 1‐mm Hg elevation in resting mean arterial blood pressure, *P*=0.806). Furthermore, multivariable regression analyses of RA versus RV parameters demonstrated that all absolute and BSA‐indexed RA volumes were positively associated with RV end‐diastolic volume index (Table S6). There was a significant inverse association between RA reservoir strain and RV volume index in the preterm‐born and term‐born patients, and RA booster strain was significantly and inversely associated with RV mass in both groups.

### Altered LA and RA Structure and Function in Adults Born Preterm Across Gestational Ages

Both extremely‐to‐very preterm‐born (<32 weeks' gestation) and moderately‐to‐late preterm‐born adults (≥32 to <37 weeks' gestation) adults had significantly smaller LV and RV end‐diastolic and stroke volume indexes than term‐born adults, as well as greater LV and RV myocardial mass indexes (Table S7). There were no statistically significant structural LV or RV differences between moderately‐to‐late preterm‐born and extremely‐to‐very preterm‐born adults, except for RV mass index, which was greater in the latter group.

Although absolute LA maximal and stroke volumes were smaller in extremely‐to‐very preterm‐born adults than in term‐born adults, absolute and indexed LA maximal, minimal, and stroke volumes were similar between moderately‐to‐late preterm‐born and term‐born adults (Table S7). There were no significant between‐group differences for strain and strain rate measurements, except for booster strain, which was reduced in the extremely‐to‐very preterm group compared with the moderately‐to‐late preterm group. Furthermore, the LA maximal volume to LV end‐diastolic volume and LA stroke volume to LV stroke volume ratios were elevated in extremely‐to‐very and moderately‐to‐late preterm‐born adults in comparison to term‐born adults. The LA‐to‐LV ratio at the end‐diastolic time point, however, was only elevated in the extremely‐to‐very preterm group.

Both absolute and indexed RA maximal, minimal, and stroke volumes were reduced in extremely‐to‐very and moderately‐to‐late preterm‐born adults compared with term‐born adults (Table S7). There were no statistically significant volumetric differences between extremely‐to‐very and moderately‐to‐late preterm‐born adults. However, there were several group differences among the RA deformation parameters. Indeed, reservoir and conduit strain were greater in extremely‐to‐very but not in moderately‐to‐late preterm‐born adults compared with term‐born adults. Booster strain values, however, were increased in the moderately‐to‐late preterm group but not in the extremely‐to‐very preterm group. In addition, RA minimal volume to RV end‐systolic volume and RA maximal volume to RV end‐systolic ratios were smaller in extremely‐to‐very preterm and moderately‐to late preterm adults than in term‐born adults. Nevertheless, there were no statistically significant differences for RA maximal volume to RV end‐diastolic volume, RA stroke volume to RV stroke volume, or RA minimal volume to RV end‐diastolic volume ratios between the groups.

## DISCUSSION

This study demonstrates that in adults born preterm, LA and RA structure and function are altered compared with those born at term. Although there were no statistically significant differences in LA volumes between the preterm‐born and term‐born adults, preterm birth was associated with increased end‐systolic and end‐diastolic LA volume to LV volume ratios. Furthermore, there were no statistically significant differences in LA function except for LA booster strain rate, which was increased in preterm‐born adults. In contrast to the LA, all measures of RA size were decreased in the preterm‐born group, including RA volumes indexed to RV volumes. The RA of preterm‐born adults also showed enhanced reservoir and booster strain values together with a global increase in strain rate, although RA stroke volume was significantly reduced in preterm‐born adults compared with term‐born adults. These structural and functional alterations in the LA and RA were observed in moderately‐to‐late preterm‐born adults, but they were most pronounced in extremely‐to‐very preterm‐born adults.

### Potential Mechanisms of LA Remodeling in Preterm‐Born Adults

Preterm birth is associated with changes in cardiovascular morphology and function that persist into adulthood.^39^, ^40^, ^41^ In addition to alterations in vascular structure and increases in blood pressure,^42^, ^43^, ^44^, ^45^ adults born preterm have smaller LV volumes and LV diastolic dysfunction.^6^, ^20^, ^46^, ^47^, ^48^ In the present study, we found that preterm‐born adults had similar LA volumes compared with term‐born adults. When indexed to LV volumes, however, the LA volumes were significantly greater in the preterm group, which could suggest a potential adaptation to morphological and functional alternations in the LV. Indeed, LV diastolic dysfunction causes elevations in LA pressure to maintain adequate LV filling. This might help explain the relative LA enlargement, which is an established surrogate for severity and chronicity of LV pressure elevation.^49^, ^50^ Furthermore, an augmented LA booster function can also act as a compensatory mechanism for impaired early filling in patients with mild diastolic dysfunction.^51^, ^52^ Although this might provide an explanation for the enhanced booster strain rate in the preterm‐born cohort, the difference is borderline statistically significant, and the absolute mean difference is small and unlikely to be of practical significance. In addition, booster strain values were not significantly different between groups, suggesting this should be interpreted with caution.

The typical relationships between LV pressure elevation and LA enlargement may not entirely apply to the preterm population. As the cardiovascular growth trajectory of preterm‐born individuals deviates from that of term‐born individuals, it should be considered that structural heart differences in preterm‐born versus term‐born adults may be caused by maturational deficits that take place in infancy, before any rise in filling pressures has occurred. Two different studies investigating preterm‐born 5‐ to 6‐year‐olds showed that the LA and LV of preterm‐born children were smaller than those of term‐born children.^53^, ^54^ Both studies investigated populations with a low mean gestational age (28.7±2.7 and 24.9±1.0 weeks) and presented findings similar to the reduced LA and LV volumes we observed for the extremely‐to‐very preterm‐born adults in our cohort compared with term‐born adults. While it would be of interest to compare the percentage difference between groups for LA and LV changes described in the present study with those seen in preterm‐born infants to gain insight into the mechanism of LA remodeling in preterm‐born individuals, LA size has not been adequately reported in preterm‐born infants. It is plausible that LA remodeling in preterm‐born individuals may have started early in life.^55^ The physiological fetal‐to‐neonatal transition involves a strong decrease in pulmonary vascular resistance following lung inflation, causing pulmonary blood flow and LA pressure to rise rapidly.^7^, ^56^ Because prematurely born neonates begin extrauterine life before the fetal circulation has matured, it is plausible that the LA of preterm‐born individuals undergo relative enlargement because of early LA pressure elevation during this critical phase of development. However, these hypotheses remain speculative. Longitudinal studies are still required to investigate the currently understudied temporal evolution in atrial and ventricular structure over time in preterm‐born compared with term‐born neonates and give a more definite answer as to which mechanisms underlie the structural changes in preterm‐born adults.

### Potential Mechanisms of RA Remodeling in Preterm‐Born Adults

We found that young adults born preterm had smaller RA volumes than those born at term, even when corrected for RV size. Preterm‐born adults show smaller RV volumes and impaired RV systolic function,^6^, ^21^, ^57^ and RV systolic dysfunction is more pronounced in those with a history of bronchopulmonary dysplasia.^58^ In addition, preterm birth is associated with pulmonary vascular disease in adulthood.^57^, ^59^, ^60^, ^61^, ^62^ Because RV dysfunction and pulmonary hypertension may lead to elevated RV pressures, a relative enlargement of the RA in preterm‐born adults could be expected. Nevertheless, previous echocardiographic studies also report associations between preterm birth and reduced RA volumes in children and adults.^63^, ^64^ As such, it seems that other factors influence RA development and remodeling in this population.

The suboptimal fetal‐to‐neonatal transition that accompanies preterm birth might partly explain the observed smaller RA volumes. Fetal cardiac development is at least partly driven by pressure and volume loading.^55^, ^65^, ^66^ When the umbilical cord is cut, the systemic vascular resistance suddenly increases, causing systemic venous return to decrease and RA pressure to drop.^67^, ^68^ Because preterm‐born individuals are exposed to high fetal RA pressures for a shorter period of time compared with term‐born individuals, their RA might experience less‐than‐physiological enlargement during this important stage of cardiac development that programs long‐term changes.^55^, ^65^, ^66^

The changes in the RA of preterm‐born adults were not limited to structure, because all measures of RA deformation except conduit strain were greater in this cohort. The enhanced reservoir and booster strain values indicate that the RA of preterm‐born adults expand more during filling and contract more during the atrial booster phase. Both of these mechanisms seem to enhance atrial stroke volume and thus might compensate for the reduced RA volumes, although indexed RV stroke volume remained lower in preterm‐born compared with term‐born adults. These findings align with previous findings of altered RV flow patterns in preterm‐born adults during exercise,^69^ as changes in RA deformation might contribute to alterations in RV flow during diastole. Additionally, the exaggerated RA booster function also corresponds to previous findings suggesting possible compensatory mechanisms for volumetric limitations in the RV of preterm‐born adults. Indeed, Barton et al.^70^ have reported that preterm‐born adults have an augmented RV contractile response when stressed under hypoxic conditions, in spite of their reduced RV volumetric reserve.^57^ As such, it seems that volumetric but not contractile reserve may be limited in the RA as well as RV of people born preterm.

### Novel Insights into the Effect of Preterm Birth on the LV and RV

Previous work suggests that preterm‐born adults born at the earliest gestations display more pronounced LV and RV alterations than those born at later gestations.^6^, ^21^, ^40^ Our current study corroborates this notion, as the extremely‐to‐very preterm‐born adults had the smallest cardiac—including RA and LA—volumes of all participants in this study. These results align with the previously proposed hypothesis of cardiopulmonary dysanapsis in preterm‐born adults.^60^ The dysanapsis hypothesis puts the idea forward that certain parts of the respiratory and cardiovascular systems in preterm‐born individuals grow and develop insufficiently from birth until adulthood and that this is a central feature underlying the cardiopulmonary functional deficits in preterm‐born individuals.^60^ Evidence from animal models^71^, ^72^, ^73^ and human histomorphological studies^74^ suggest that preterm birth may cause a premature interruption of the intrauterine cardiomyocyte hyperplasia and subsequent lower cardiomyocyte endowment, which may underlie the volumetric impairments in preterm‐born individuals, especially those born at the earliest gestations.^75^

Although preterm‐born adults more often demonstrate functional LV and RV deficits in comparison with term‐born adults, previous work has shown that LV ejection fraction remains preserved at rest.^20^, ^27^, ^47^ In our current study, however, LV ejection fraction was significantly lower in preterm‐born versus term‐born adults (−1.6%, *P*=0.002). It is plausible that this may be because of the relatively large sample size of our current study, which is the largest to date to use individual patient data to investigate cardiac changes in preterm‐born adults. The combination of the sample size and use of CMR imaging, which is considered the gold standard measure for LV ejection fraction, provides greater power to detect small differences and reduces the likelihood of type II errors.^76^ Nevertheless, these observed statistical differences in LV ejection fraction are clinically modest and should be investigated further, especially as part of longitudinal research programs.

### Clinical Implications of Atrial Remodeling in Preterm‐Born Adults

Preterm birth is associated with increased rates of cardiovascular disease and mortality in early adulthood.^2^, ^3^, ^4^, ^5^, ^77^, ^78^ Even though epidemiological data on the associations of prematurity on cardiovascular outcomes are not yet available in the elderly population, the identification of preterm birth as a significant cardiovascular risk factor offers an opportunity for early screening and prevention strategies.^39^, ^40^ Previously proposed screening considerations include lifestyle assessment and blood pressure screening for all preterm‐born adults, as well as early exercise testing, pulmonary function assessment, and echocardiographic imaging for symptomatic preterm‐born adults or preterm‐born adults with a high‐risk birth history (eg, intrauterine growth restriction, preeclampsia, and bronchopulmonary dysplasia).^40^ In this context, there could be a place for the evaluation of LA and RA structure and function. Indeed, atrial remodeling has been shown to independently predict cardiovascular outcomes,^79^, ^80^ including heart failure,^13^, ^15^ stroke,^11^, ^12^ and cardiovascular death,^11^ all of which occur more often and earlier in adults born preterm. For optimal clinical implementation of LA and RA screening, its applicability has yet to be validated in the context of the preterm heart, and appropriate reference ranges must be established for LA and RA volumes, strain values, and strain rate values of preterm‐born neonates, children, and adults. However, screening for LA and RA irregularities and measurements over time could ultimately become a valuable component of an integrated screening approach to aid in modification of lifetime cardiovascular risk in preterm‐born individuals.^40^

### Strengths and Limitations

In this study, we report findings from a large cohort of participants who underwent CMR imaging, the current gold standard technique for noninvasive evaluation of heart function and volumes. For the first time, we evaluated the relationship between gestational age and bi‐atrial phenotype using CMR imaging, resulting in the largest preterm adult cohort to date with comprehensive characterization of bi‐atrial volume, strain, and strain rate values. However, this study has some limitations. First, because of the novelty of the methodology for the atrial strain and strain rate analyses, there is a lack of standardization across different analysis systems. However, we used a previously validated method^23^ and all measures of intraobserver and interobserver variability were within an acceptable range based on similar imaging methods.^23^, ^24^, ^37^, ^38^ In addition, measures of LA and RA size in the term‐born cohort were similar to previously published CMR reference values in healthy participants.^81^ Second, our cohort comprised only a small number of participants who were born extremely preterm (<28 weeks' gestation). Because of this small sample size, we could not perform subgroup analyses between these participants and participants born at a later gestational age. Further work is needed to explore atrial structure and function in those born at the earliest gestations. Third, because the cohort included in the present study was primarily White, we could not explore whether the associations between preterm birth and atrial parameters were similar across different races, highlighting the need for further research in this area. Fourth, we did not have complete information on all perinatal complications (eg, bronchopulmonary dysplasia, neonatal infections, and hypertensive pregnancies) and interventions (eg, assisted ventilation, steroid therapy, and oxygen supplementation) because of the retrospective perinatal data collection in a proportion of our cohort. In addition, we did not measure pulmonary function or vasculature in all participants. Because preterm‐born adults more often have pulmonary dysfunction and pulmonary vascular disease,^57^, ^59^ it would be of interest to know how the RA alterations relate to the pulmonary vasculature in this population. Further studies will be needed to examine the impacts of these factors. In addition, considering the previous evidence suggesting that maternal milk may be protective against potentially adverse cardiac remodeling observed in preterm‐born individuals,^82^, ^83^, ^84^ it would be of interest to investigate this in future studies. Nevertheless, our data on gestational age and birth weight were complete and allowed us to determine that degree of prematurity may be an important determinant of atrial structure and function in young adults. Fifth, the percentage of preterm‐born adults born small for gestational age in our cohort is low compared with the general population, which is important to note given the associated independent cardiovascular risk of being born small for gestational age.^85^ Nevertheless, our results may better reflect the direct association with preterm birth and gestational age rather than the confounding effects of small for gestational age.

## CONCLUSIONS

Young adults born preterm show distinct changes in cardiac structure and function compared with those born at term. In this study, we demonstrate that the LA of preterm‐born adults are similar in size to those of term‐born adults, while the RA are smaller and show enhanced strain and strain rate. These structural and functional atrial alterations might represent compensatory behavior in response to the cardiopulmonary impairments in preterm‐born adults. Further research into the mechanistic pathways and prognostic utility of the altered cardiac—including atrial—phenotype in preterm‐born adults is warranted and could form an important component of preventative approaches for cardiovascular disease in this population.

## Sources of Funding

The British Heart Foundation (BHF) and the Wellcome Trust provided study funding. The authors acknowledge support from the Oxford BHF Centre for Research Excellence and National Institute for Health Research (NIHR) Oxford Biomedical Research Centre. Dr Lewandowski is funded by a British Heart Foundation Intermediate Research Fellowship (FS/18/3/33292).

## Disclosures

None.

## Supporting information

Tables S1–S7
Figures S1–S2
