# Supplementary material for: Differing Impact of Preterm Birth on the Right and Left Atria in Adulthood
Source: J Am Heart Assoc. 2022 Dec 6;11(23):e027305. doi: 10.1161/JAHA.122.027305 (PMC9851437; doi:10.1161/JAHA.122.027305)
Supplement: Supplementary file 1 — Tables S1–S7 Figures S1–S2 [file JAH3-11-e027305-s001.pdf]

SUPPLEMENTAL MATERIAL

Table S1. Intraobserver and Interobserver Coefficients of Variation for Left and Right Atrial Parameters.

	Intraobserver CoV, %	Interobserver CoV, %
**Left atrium**		
Minimal LA volume	1.15	16.75
Maximal LA volume	2.15	11.18
LA stroke volume	3.88	8.9
LA ejection fraction	1.74	7.72
LA reservoir strain	3.61	12.24
LA conduit strain	2.42	13.99
LA booster strain	5.83	16.6
LA reservoir strain rate	12.61	11.12
LA conduit strain rate	5.21	22.62
LA booster strain rate	13.37	27.77
**Right atrium**		
Minimal RA volume	3.59	17.01
Maximal RA volume	2.03	11.11
RA stroke volume	6.91	21.65
RA ejection fraction	4.49	15.37
RA reservoir strain	4.82	11.94
RA conduit strain	4.82	13.37
RA booster strain	12.61	20.06
RA reservoir strain rate	7.80	20.89
RA conduit strain rate	13.33	13.97
RA booster strain rate	23.85	24.42

CoV, coefficient of variation; LA, left atrial; RA, right atrial.

Table S2. Left and Right Atrial Structure and Function in Preterm-Born and Term-Born Male and Female Participants.

	Male participants (n=227)			Female participants (n=239)		
	**Preterm-born adults (n=91)**	**Term-born adults (n=136)**	**P value**	**Preterm-born adults (n=109)**	**Term-born adults (n=130)**	**P value**
**Left atrium**						
Booster strain rate, 1/s	-0.82 ± 0.28	-0.66 ± 0.30	**0.004**	-0.71 ± 0.26	-0.68 ± 0.24	0.539
LA max. volume to LV end-diastolic volume ratio	0.44 ± 0.10	0.40 ± 0.10	**<0.001**	0.45 ± 0.08	0.43 ± 0.09	**0.034**
LA stroke volume to LV stroke volume ratio	0.45 ± 0.11	0.39 ± 0.09	**<0.001**	0.46 ± 0.09	0.44 ± 0.09	**0.014**
LA min. volume to LV end-diastolic volume ratio	0.16 ± 0.05	0.15 ± 0.06	0.053	0.16 ± 0.04	0.14 ± 0.04	**0.031**
**Right atrium**						
Max. volume, mL	68.5 ± 19.5	82.8 ± 19.6	**<0.001**	51.7 ± 14.5	63.2 ± 15.1	**<0.001**
Min. volume, mL	35.6 ± 11.8	44.3 ± 13.4	**<0.001**	25.4 ± 8.6	32.0 ± 9.5	**<0.001**
Stroke volume, mL	32.8 ± 12.1	38.5 ± 11.6	**<0.001**	26.3 ± 9.3	31.2 ± 8?8	**<0.001**
Max. volume index, mL/m²	35.4 ± 9.1	41.9 ± 9.6	**<0.001**	30.6 ± 7.5	36.4 ± 7.5	**<0.001**
Min. volume index, mL/m²	18.4 ± 5.8	22.4 ± 6.6	**<0.001**	15.0 ± 4.5	18.4 ± 4.8	**<0.001**
Stroke volume index, mL/m²	17.0 ± 5.8	19.5 ± 5.7	**<0.001**	15.6 ± 5.2	17.9 ± 4.7	**<0.001**
Reservoir strain, %	17.9 ± 3.9	16.9 ± 3.6	0.077	20.8 ± 4.3	19.9 ± 4.1	0.080
Booster strain, %	5.6 ± 2.4	5.1 ± 2.5	0.111	5.8 ± 2.7	5.2 ± 2.7	0.069
Reservoir strain rate, 1/s	0.95 ± 0.27	0.89 ± 0.23	0.088	1.05 ± 0.27	0.97 ± 0.21	**0.022**
Conduit strain rate, 1/s	-1.09 ± 0.35	-1.00 ± 0.29	**0.046**	-1.30 ± 0.42	-1.24 ± 0.35	0.283
Booster strain rate, 1/s	-0.71 ± 0.24	-0.67 ± 0.26	0.379	-0.75 ± 0.31	-0.66 ± 0.29	**0.108**

RA max. volume to RV end-diastolic volume ratio	0.42 ± 0.10	0.45 ± 0.10	0.109	0.41 ± 0.09	0.44 ± 0.09	0.053
RA min. volume to RV end-systolic volume ratio	0.52 ± 0.17	0.58 ± 0.20	**0.011**	0.48 ± 0.16	0.58 ± 0.17	**<0.001**
RA max. volume to RV end-systolic volume ratio	0.99 ± 0.28	1.09 ± 0.29	**0.014**	0.98 ± 0.29	1.15 ± 0.33	**<0.001**
RA min. volume to RV end-diastolic volume ratio	0.11 ± 0.03	0.12 ± 0.03	0.211	0.12 ± 0.03	0.13 ± 0.03	0.071

Group characteristics presented as mean ± SD. P values represent between-group comparisons that were adjusted for differing age distributions using multivariable linear regression. P values in bold indicate statistical significance (P<0.05). LA, left atrial; LV, left ventricular; RA, right atrial; RV, right ventricular.

Table S3. Multivariable Linear Regression of Left and Right Atrial Structural and Functional Parameters Versus Gestational Age and Birth Weight.

	Gestational age (in weeks)		Birth weight (as z score)	
	B (95% CI)	P value	B (95% CI)	P value
**LA parameters**				
Booster strain rate, 1/s	0.008 (0.000; 0.015)	0.052	0.020 (-0.013; 0.053)	0.229
LA max. volume to LV end-diastolic volume ratio	-0.003 (-0.005; -0.001)	**<0.001**	-0.005 (-0.014; 0.003)	0.223
LA stroke volume to LV stroke volume ratio	-0.004 (-0.006; -0.002)	**<0.001**	0.000 (-0.009; 0.009)	0.977
LA min. volume to LV end-diastolic volume ratio	-0.001 (-0.002; 0.000)	**0.004**	-0.004 (-0.008; 0.001)	0.131
**RA parameters**				
Max. volume, mL	1.518 (1.129; 1.907)	**<0.001**	1.104 (-0.720; 2.927)	0.236
Min. volume, mL	0.892 (0.645; 1.139)	**<0.001**	0.162 (-0.999; 1.323)	0.784
Stroke volume, mL	0.626 (0.409; 0.844)	**<0.001**	0.941 (-0.08; 1.962)	0.071
Max. volume index, mL/m²	0.674 (0.497; 0.850)	**<0.001**	0.312 (-0.517; 1.142)	0.461
Min. volume index, mL/m²	0.406 (0.289; 0.523)	**<0.001**	-0.059 (-0.607; 0.488)	0.832
Stroke volume index, mL/m²	0.268 (0.161; 0.375)	**<0.001**	0.372 (-0.130; 0.874)	0.147
Reservoir strain, %	-0.165 (-0.256; -0.075)	**<0.001**	0.094 (-0.321; 0.509)	0.656
Booster strain, %	-0.060 (-0.115; -0.006)	**0.031**	0.156 (-0.094; 0.407)	0.222
Reservoir strain rate, 1/s	-0.008 (-0.013; -0.003)	**0.003**	0.011 (-0.013; 0.036)	0.357
Conduit strain rate, 1/s	0.012 (0.004; 0.020)	**0.003**	-0.021 (-0.057; 0.015)	0.262
Booster strain rate, 1/s	0.009 (0.001; 0.017)	**0.037**	-0.024 (-0.058; 0.009)	0.156
RA max. volume to RV end-diastolic volume ratio	0.002 (0.00; 0.004)	**0.022**	0.005 (-0.004; 0.014)	0.284
RA min. volume to RV end-systolic volume ratio	0.008 (0.005; 0.012)	**<0.001**	0.001 (-0.016; 0.017)	0.934
RA max. volume to RV end-systolic volume ratio	0.013 (0.007; 0.019)	**<0.001**	0.013 (-0.015; 0.041)	0.367
RA min. volume to RV end-diastolic volume ratio	0.000 (0.000; 0.001)	0.153	-0.001 (-0.004; 0.002)	0.540

B is the unstandardised regression coefficient and represents the difference in the indicated LA or RA variable per 1-week elevation in gestational age or per 1-z score elevation in birth weight, respectively. P values in bold indicate statistical significance ($P<0.05$). CI, confidence interval; LA, left atrial; RA, right atrial.

Table S4. Multivariable Linear Regression of Left and Right Atrial Structural and Functional Parameters Versus Cardiovascular Risk Factors in Preterm-Born and Term-Born Adults.

	Group	BMI (in kg/m²)		Resting mean arterial pressure (in mmHg)		Smoker (yes vs no)	
		**B (95% CI)**	**P value**	**B (95% CI)**	**P value**	**B (95% CI)**	**P value**
**Left atrium**							
LA booster strain rate, 1/s	Term	-0.007 (-0.020; 0.007)	0.337	0.000 (-0.005; 0.005)	0.978	-0.121 (-0.305; 0.063)	0.201
	Preterm	-0.017 (-0.033; -0.001)	**0.037**	-0.001 (-0.007; 0.005)	0.651	-0.033 (-0.568; 0.503)	0.905
LA max. volume to LV end-diastolic volume ratio	Term	0.002 (-0.002; 0.005)	0.356	0.001 (-0.001; 0.002)	0.268	0.088 (0.048; 0.127)	**<0.001**
	Preterm	0.002 (-0.002; 0.006)	0.284	0.000 (-0.002; 0.002)	0.975	-0.034 (-0.076; 0.008)	0.117
LA stroke volume to LV stroke volume ratio	Term	0.000 (-0.004; 0.003)	0.908	0.000 (-0.001; 0.001)	0.895	0.062 (0.023; 0.101)	**0.002**
	Preterm	0.001 (-0.003; 0.005)	0.683	0.000 (-0.002; 0.002)	0.909	-0.071 (-0.115; -0.026)	**0.002**
LA min. volume to LV end-diastolic volume ratio	Term	0.001 (-0.001; 0.003)	0.393	0.000 (-0.001; 0.001)	0.589	0.049 (0.028; 0.069)	**<0.001**
	Preterm	0.001 (-0.001; 0.002)	0.520	0.000 (-0.001; 0.001)	0.752	0.006 (-0.016; 0.028)	0.598
**Right atrium**							
RA max. volume, mL	Term	1.435 (0.691; 2.180)	**<0.001**	-0.058 (-0.349; 0.232)	0.695	-1.171 (-9.521; 7.179)	0.784
	Preterm	1.773 (1.075; 2.472)	**<0.001**	0.055 (-0.255; 0.366)	0.729	-2.726 (-10.963; 5.512)	0.517

RA min. volume, mL	Term	0.877 (0.39; 1.364)	**<0.001**	-0.086 (-0.276; 0.104)	0.378	0.128 (-5.337; 5.593)	0.964
	Preterm	0.868 (0.446; 1.290)	**<0.001**	0.180 (-0.008; 0.368)	0.062	1.241 (-3.739; 6.220)	0.626
RA stroke volume, mL	Term	0.558 (0.144; 0.972)	**0.009**	0.028 (-0.134; 0.189)	0.738	-1.299 (-5.938; 3.341)	0.584
	Preterm	0.906 (0.487; 1.325)	**<0.001**	-0.125 (-0.311; 0.061)	0.191	-3.966 (-8.909; 0.977)	0.117
RA max. volume index, mL/m²	Term	0.105 (-0.237; 0.447)	0.548	-0.059 (-0.193; 0.074)	0.384	-0.767 (-4.603; 3.070)	0.696
	Preterm	0.503 (0.172; 0.833)	0.003	0.002 (-0.145; 0.149)	0.977	-1.576 (-5.477; 2.324)	0.429
RA min. volume index, mL/m²	Term	0.120 (-0.110; 0.350)	0.306	-0.057 (-0.146; 0.033)	0.216	-0.008 (-2.585; 2.569)	0.995
	Preterm	0.238 (0.031; 0.444)	0.025	0.089 (-0.003; 0.181)	0.059	0.563 (-1.869; 2.994)	0.651
RA stroke volume index, mL/m²	Term	-0.015 (-0.219; 0.189)	0.884	-0.003 (-0.082; 0.077)	0.947	-0.759 (-3.044; 1.527)	0.516
	Preterm	0.265 (0.052; 0.479)	**0.016**	-0.087 (-0.182; 0.008)	0.076	-2.139 (-4.661; 0.384)	0.098
RA reservoir strain, %	Term	-0.079 (-0.240; 0.083)	0.341	-0.067 (-0.128; -0.006)	**0.032**	0.502 (-1.244; 2.247)	0.574
	Preterm	-0.022 (-0.224; 0.180)	0.834	-0.099 (-0.18; -0.019)	**0.017**	-1.054 (-3.255; 1.146)	0.349
RA booster strain, %	Term	0.064 (-0.035; 0.162)	0.206	0.040 (0.002; 0.077)	**0.038**	-1.646 (-2.713; -0.579)	**0.003**
	Preterm	0.009 (-0.11; 0.129)	0.881	0.006 (-0.042; 0.054)	0.806	-1.922 (-3.225; -0.619)	**0.004**
RA reservoir strain rate, 1/s	Term	-0.004 (-0.013; 0.005)	0.370	-0.002 (-0.005; 0.002)	0.316	-0.001 (-0.096; 0.094)	0.986
	Preterm	-0.017 (-0.030; -0.004)	**0.009**	-0.003 (-0.008; 0.002)	0.217	-0.124 (-0.262; 0.014)	0.080

RA conduit strain rate, 1/s	Term	0.024 (0.011; 0.037)	**<0.001**	0.004 (-0.001; 0.009)	0.086	-0.109 (-0.250; 0.031)	0.129
	Preterm	0.018 (0.001; 0.036)	**0.045**	0.004 (-0.003; 0.011)	0.257	0.047 (-0.147; 0.242)	0.634
RA booster strain rate, 1/s	Term	-0.001 (-0.014; 0.013)	0.924	-0.007 (-0.012; -0.002)	**0.005**	0.161 (-0.019; 0.341)	0.082
	Preterm	0.005 (-0.013; 0.023)	0.594	0.002 (-0.004; 0.008)	0.510	-0.239 (-0.807; 0.329)	0.411
RA max. volume to RV end-diastolic volume ratio	Term	0.002 (-0.002; 0.005)	0.311	0.000 (-0.001; 0.001)	0.875	0.002 (-0.038; 0.042)	0.922
	Preterm	0.006 (0.002; 0.010)	**0.001**	0.000 (-0.001; 0.002)	0.791	-0.027 (-0.071; 0.017)	0.231
RA min. volume to RV end-systolic volume ratio	Term	0.006 (-0.001; 0.013)	0.099	0.000 (-0.003; 0.003)	0.926	0.052 (-0.025; 0.13)	0.189
	Preterm	0.012 (0.006; 0.018)	**<0.001**	0.004 (0.001; 0.007)	**0.006**	0.027 (-0.045; 0.099)	0.470
RA max. volume to RV end-systolic volume ratio	Term	0.007 (-0.004; 0.019)	0.223	0.001 (-0.003; 0.006)	0.568	0.069 (-0.063; 0.202)	0.306
	Preterm	0.024 (0.013; 0.034)	**<0.001**	0.002 (-0.003; 0.006)	0.460	-0.042 (-0.166; 0.082)	0.509
RA min. volume to RV end-diastolic volume ratio	Term	-0.001 (-0.002; 0.000)	**0.050**	0.000 (-0.001; 0.000)	0.374	0.004 (-0.010; 0.017)	0.621
	Preterm	0.000 (-0.001; 0.001)	0.996	0.001 (0.000; 0.001)	**0.031**	0.003 (-0.012; 0.018)	0.741

B is the unstandardised regression coefficient and represents the difference in the indicated LA or RA variable per 1-unit elevation in BMI (in kg/m²), mean arterial blood pressure (in mmHg), or smoking status (yes [1] vs no [0]). P values in bold indicate statistical significance (P<0.05). BMI, body mass index; CI, confidence interval; LA, left atrial; LV, left ventricular; RA, right atrial; RV, right ventricular.

Table S5. Multivariable Linear Regression of Left Atrial Structural and Functional Parameters Versus Left Ventricular Structural and Functional Parameters in Preterm-Born and Term-Born Adults.

		LV end-diastolic volume index (in mL/m²)		LV mass index (in g/m²)		LV ejection fraction (in %)		Heart rate (in b.p.m.)	
	**Group**	**B (95% CI)**	**P value**	**B (95% CI)**	**P value**	**B (95% CI)**	**P value**	**B (95% CI)**	**P value**
LA booster strain rate, 1/s	Term	-0.001 (-0.005; 0.003)	0.599	0.001 (-0.005; 0.007)	0.803	-0.004 (-0.012; 0.005)	0.377	-0.005 (-0.010; -0.001)	**0.020**
	Preterm	0.000 (-0.007; 0.007)	0.928	-0.002 (-0.010; 0.006)	0.708	-0.002 (-0.011; 0.007)	0.672	-0.011 (-0.017; -0.005)	**<0.001**
LA max. volume to LV end-diastolic volume ratio	Term	-0.002 (-0.003; 0.000)	**0.009**	0.001 (0.000; 0.002)	0.113	0.004 (0.001; 0.006)	**0.001**	0.001 (0.000; 0.002)	0.209
	Preterm	-0.002 (-0.004; -0.001)	**0.009**	0.001 (0.000; 0.003)	0.070	0.002 (0.000; 0.004)	0.082	-0.001 (-0.002; 0.001)	0.375
LA stroke volume to LV stroke volume ratio	Term	-0.001 (-0.002; 0.000)	**0.021**	0.000 (-0.001; 0.001)	0.982	-0.001 (-0.003; 0.001)	0.382	0.000 (-0.001; 0.001)	0.665
	Preterm	-0.001 (-0.003; 0.000)	0.100	-0.001 (-0.002; 0.001)	0.439	-0.003 (-0.006; -0.001)	**0.007**	-0.001 (-0.002; 0.001)	0.240
LA min. volume to LV end-diastolic volume ratio	Term	-0.001 (-0.001; 0.000)	**0.047**	0.001 (0.000; 0.002)	**0.004**	0.001 (0.000; 0.002)	0.239	0.000 (0.000; 0.001)	0.208
	Preterm	-0.001 (-0.002; 0.000)	**0.002**	0.002 (0.001; 0.003)	**<0.001**	0.000 (-0.001; 0.001)	0.681	0.000 (-0.001; 0.000)	0.655

B is the unstandardised regression coefficient and represents the difference in the indicated LA variable per 1-unit elevation in LV end-diastolic volume index (in mL/m²), LV mass index (in g/m²), LV ejection fraction (in %) or heart rate (in b.p.m.). P values in bold indicate statistical significance (P<0.05). CI, confidence interval; LA, left atrial; LV, left ventricular.

Table S6. Multivariable Linear Regression of Right Atrial Structural and Functional Parameters Versus Right Ventricular Structural and Functional Parameters in Preterm-Born and Term-Born Adults.

		RV end-diastolic volume index (in mL/m²)		RV mass index (in g/m²)		RV ejection fraction (in %)		Heart rate (in b.p.m.)	
	**Group**	**B (95% CI)**	**P value**	**B (95% CI)**	**P value**	**B (95% CI)**	**P value**	**B (95% CI)**	**P value**
RA max. volume, mL	Term	0.610 (0.416; 0.804)	**<0.001**	0.163 (-0.659; 0.985)	0.698	-0.171 (-0.574; 0.233)	0.408	-0.209 (-0.443; 0.026)	0.083
	Preterm	0.752 (0.551; 0.952)	**<0.001**	-0.002 (-0.694; 0.690)	0.996	0.032 (-0.293; 0.356)	0.849	-0.020 (-0.253; 0.213)	0.866
RA min. volume, mL	Term	0.312 (0.183; 0.441)	**<0.001**	0.639 (0.093; 1.184)	**0.023**	-0.046 (-0.314; 0.221)	0.734	-0.142 (-0.298; 0.014)	0.075
	Preterm	0.434 (0.313; 0.556)	**<0.001**	0.044 (-0.377; 0.464)	0.839	0.007 (-0.190; 0.204)	0.945	-0.011 (-0.152; 0.131)	0.884
RA stroke volume, mL	Term	0.298 (0.183; 0.413)	**<0.001**	-0.475 (-0.961; 0.010)	0.056	-0.124 (-0.362; 0.114)	0.308	-0.067 (-0.205; 0.072)	0.347
	Preterm	0.317 (0.188; 0.446)	**<0.001**	-0.046 (-0.491; 0.400)	0.841	0.025 (-0.184; 0.233)	0.817	-0.010 (-0.160; 0.141)	0.901
RA max. volume index, mL/m²	Term	0.251 (0.163; 0.34)	**<0.001**	0.169 (-0.205; 0.544)	0.377	-0.054 (-0.238; 0.130)	0.565	-0.112 (-0.219; -0.005)	**0.041**
	Preterm	0.295 (0.201; 0.389)	**<0.001**	0.112 (-0.212; 0.435)	0.500	0.037 (-0.115; 0.188)	0.633	-0.022 (-0.132; 0.087)	0.687
RA min. volume index, mL/m²	Term	0.131 (0.071; 0.192)	**<0.001**	0.355 (0.100; 0.611)	**0.007**	-0.013 (-0.139; 0.112)	0.837	-0.074 (-0.147; -0.001)	**0.048**
	Preterm	0.181 (0.122; 0.240)	**<0.001**	0.089 (-0.114; 0.292)	0.393	0.013 (-0.082; 0.109)	0.784	-0.011 (-0.079; 0.058)	0.754
RA stroke volume index, mL/m²	Term	0.120 (0.063; 0.177)	**<0.001**	-0.186 (-0.428; 0.055)	0.131	-0.041 (-0.159; 0.077)	0.499	-0.038 (-0.107; 0.031)	0.278
	Preterm	0.115 (0.048; 0.181)	**<0.001**	0.023 (-0.206; 0.251)	0.845	0.024 (-0.083; 0.131)	0.667	-0.012 (-0.089; 0.066)	0.770

RA reservoir strain, %	Term	-0.065 (-0.112; -0.018)	**0.007**	0.019 (-0.172; 0.210)	0.848	0.127 (0.030; 0.223)	**0.011**	-0.039 (-0.096; 0.018)	0.183
	Preterm	-0.104 (-0.165; -0.043)	**0.001**	0.044 (-0.157; 0.245)	0.669	0.004 (-0.088; 0.096)	0.933	0.035 (-0.044; 0.114)	0.384
RA booster strain, %	Term	0.018 (-0.012; 0.047)	0.242	-0.229 (-0.349; -0.109)	**<0.001**	0.019 (-0.041; 0.080)	0.531	0.018 (-0.018; 0.054)	0.320
	Preterm	0.014 (-0.021; 0.050)	0.429	-0.205 (-0.323; -0.087)	**<0.001**	-0.032 (-0.086; 0.022)	0.250	0.032 (-0.015; 0.078)	0.181
RA reservoir strain rate, 1/s	Term	-0.002 (-0.004; 0.001)	0.150	-0.007 (-0.018; 0.003)	0.159	0.005 (-0.001; 0.010)	0.082	0.003 (-0.001; 0.006)	0.116
	Preterm	-0.005 (-0.008; -0.001)	**0.027**	0.000 (-0.013; 0.013)	0.966	-0.002 (-0.007; 0.004)	0.606	-0.001 (-0.006; 0.004)	0.751
RA conduit strain rate, 1/s	Term	0.008 (0.004; 0.012)	**<0.001**	-0.026 (-0.042; -0.010)	**0.002**	-0.008 (-0.016; 0.000)	0.064	0.000 (-0.005; 0.005)	0.974
	Preterm	0.006 (0.000; 0.012)	**0.039**	-0.010 (-0.029; 0.009)	0.292	0.003 (-0.006; 0.011)	0.511	-0.006 (-0.014; 0.001)	0.099
RA booster strain rate, 1/s	Term	-0.003 (-0.006; 0.001)	0.19	0.018 (-0.003; 0.039)	0.091	-0.010 (-0.017; -0.003)	**0.007**	-0.011 (-0.015; -0.006)	**<0.001**
	Preterm	-0.004 (-0.009; 0.001)	0.132	0.019 (0.001; 0.038)	0.041	-0.003 (-0.011; 0.004)	0.399	-0.012 (-0.019; -0.006)	**<0.001**
RA max. volume to RV end-diastolic volume ratio	Term	-0.002 (-0.003; -0.001)	**<0.001**	0.002 (-0.002; 0.006)	0.366	0.000 (-0.002; 0.002)	0.743	-0.001 (-0.003; 0.000)	**0.036**
	Preterm	-0.001 (-0.003; 0.000)	**0.032**	0.001 (-0.003; 0.005)	0.658	0.000 (-0.002; 0.002)	0.669	0.000 (-0.002; 0.001)	0.852
RA min. volume to RV end-systolic volume ratio	Term	-0.003 (-0.004; -0.001)	**0.004**	0.009 (0.001; 0.016)	**0.025**	0.015 (0.012; 0.019)	**<0.001**	-0.002 (-0.004; 0.000)	0.082
	Preterm	-0.001 (-0.002; 0.001)	0.456	0.002 (-0.004; 0.008)	0.544	0.012 (0.009; 0.015)	**<0.001**	0.000 (-0.002; 0.002)	0.981
RA max. volume to RV end-systolic volume ratio	Term	-0.005 (-0.008; -0.003)	**<0.001**	0.005 (-0.007; 0.016)	0.438	0.031 (0.025; 0.036)	**<0.001**	-0.003 (-0.006; 0.000)	0.078
	Preterm	-0.004 (-0.007; -0.001)	**0.008**	0.004 (-0.005; 0.014)	0.379	0.025 (0.020; 0.029)	**<0.001**	-0.001 (-0.004; 0.002)	0.645

RA min. volume to RV end-diastolic volume ratio	Term	-0.001 (-0.001; 0.000)	**<0.001**	0.002 (0.001; 0.004)	**0.007**	0.000 (-0.001; 0.001)	0.982	0.000 (-0.001; 0.000)	0.050
	Preterm	0.000 (-0.001; 0.000)	**0.024**	0.001 (-0.001; 0.002)	0.318	0.000 (-0.001; 0.001)	0.795	0.000 (0.000; 0.000)	0.976

B is the unstandardised regression coefficient and represents the difference in the indicated RA variable per 1-unit elevation in LV end-diastolic volume index (in mL/m²), RV mass index (in g/m²), RV ejection fraction (in %) or heart rate (in b.p.m.). P values in bold indicate statistical significance (P<0.05). CI, confidence interval; RA, right atrial; RV, right ventricular.

Table S7. Left and Right Heart Structure and Function in Extremely-to-Very Preterm-Born, Moderately-to-Late Preterm-Born and Term-Born Adults.

	<32 weeks (n=95)	≥32 to <37 weeks (n=105)	≥37 weeks (n=266)	P_1	P_2	P_3
**LV structure and function**						
End-diastolic volume index, mL/m²	71.2 ± 8.9	74.2 ± 11.8	81.0 ± 12.6	0.017	**<0.001**	**<0.001**
End-systolic volume index, mL/m²	26.1 ± 5.3	27.7 ± 6.1	28.8 ± 7.1	0.021	0.206	**<0.001**
Stroke volume index, mL/m²	45.1 ± 6.7	46.5 ± 8.4	52.2 ± 8.2	0.122	**<0.001**	**<0.001**
Myocardial mass index, g/m²	66.1 ± 11.1	62.9 ± 9.3	55.6 ± 9.7	0.042	**<0.001**	**<0.001**
Ejection fraction, %	63.5 ± 5.7	62.7 ± 5.5	64.7 ± 5.2	0.326	**0.001**	0.068
**RV structure and function**						
End-diastolic volume index, mL/m²	76.8 ± 11.5	80.6 ± 14.7	89.2 ± 14.6	0.011	**<0.001**	**<0.001**
End-systolic volume index, mL/m²	33.6 ± 7.1	34.9 ± 8.3	36.3 ± 8.9	0.107	0.258	**0.004**
Stroke volume index, mL/m²	43.2 ± 9.0	45.7 ± 10.1	52.9 ± 8.2	0.033	**<0.001**	**<0.001**
Myocardial mass index, g/m²	23.9 ± 3.8	21.0 ± 3.3	19.4 ± 3.1	**<0.001**	**<0.001**	**<0.001**
Ejection fraction, %	56.1 ± 7.4	56.6 ± 6.9	59.6 ± 5.5	0.598	**<0.001**	**<0.001**
**LA structure and function**						
Max. volume, mL	56.6 ± 13.5	60.1 ± 18.2	61.9 ± 17.6	0.047	0.759	**0.008**
Min. volume, mL	21.0 ± 6.7	21.1 ± 8.7	22.3 ± 8.8	0.602	0.537	0.206
Stroke volume, mL	35.7 ± 9.0	39.0 ± 10.9	39.6 ± 10.6	**0.006**	0.989	**0.001**
Max. volume index, mL/m²	32.0 ± 7.1	32.9 ± 8.1	33.3 ± 8.6	0.325	0.876	0.215
Min. volume index, mL/m²	11.8 ± 3.5	11.5 ± 4.0	11.9 ± 4.4	0.671	0.552	0.872
Stroke volume index, mL/m²	20.2 ± 4.9	21.4 ± 5.0	21.4 ± 5.3	0.065	0.821	0.066
Ejection fraction, %	63.2 ± 7.1	65.6 ± 6.0	64.8 ± 7.2	0.014	0.374	0.065
Reservoir strain, %	19.8 ± 3.2	20.1 ± 2.8	20.1 ± 3.2	0.856	0.491	0.416
Conduit strain, %	15.7 ± 2.9	15.2 ± 2.7	15.7 ± 3.0	0.104	0.027	0.821
Booster strain, %	5.4 ± 2.0	6.4 ± 2.0	5.8 ± 1.9	**0.001**	0.014	0.107
Reservoir strain rate, 1/s	0.89 ± 0.17	0.90 ± 0.16	0.92 ± 0.19	0.910	0.127	0.137
Conduit strain rate, 1/s	-1.55 ± 0.34	-1.54 ± 0.36	-1.50 ± 0.72	0.768	0.854	0.650
Booster strain rate, 1/s	-0.73 ± 0.28	-0.76 ± 0.27	-0.67 ± 0.27	0.627	0.017	0.238

LA max. volume to LV end-diastolic volume ratio	0.45 ± 0.09	0.44 ± 0.09	0.41 ± 0.96	0.626	**0.004**	**<0.001**
LA min. volume to LV end-systolic volume ratio	0.47 ± 0.16	0.42 ± 0.15	0.43 ± 0.17	0.055	0.808	0.063
LA stroke volume to LV stroke volume ratio	0.45 ± 0.10	0.47 ± 0.10	0.41 ± 0.09	0.271	**<0.001**	**<0.001**
LA max. volume to LV end-systolic volume ratio	1.27 ± 0.36	1.22 ± 0.33	1.21 ± 0.38	0.331	0.706	0.152
LA min. volume to LV end-diastolic volume ratio	0.17 ± 0.05	0.15 ± 0.05	0.15 ± 0.05	0.093	0.168	**0.001**
**RA structure and function**						
Max. volume, mL	57.6 ± 17.9	61.0 ± 19.7	73.2 ± 20.1	0.046	**<0.001**	**<0.001**
Min. volume, mL	28.9 ± 10.8	31.1 ± 11.8	38.3 ± 13.1	0.035	**<0.001**	**<0.001**
Stroke volume, mL	28.7 ± 11.2	29.9 ± 11.1	34.9 ± 10.9	0.247	**<0.001**	**<0.001**
Max. volume index, mL/m²	32.2 ± 8.7	33.3 ± 8.5	39.2 ± 9.1	0.221	**<0.001**	**<0.001**
Min. volume index, mL/m²	16.2 ± 5.4	16.9 ± 5.4	20.4 ± 6.1	0.165	**<0.001**	**<0.001**
Stroke volume index, mL/m²	16.1 ± 5.9	16.4 ± 5.2	18.7 ± 5.3	0.591	**<0.001**	**<0.001**
Ejection fraction, %	49.4 ± 10.6	49.3 ± 10.4	48.0 ± 8.8	0.779	0.389	0.248
Reservoir strain, %	20.0 ± 4.2	19.1 ± 4.5	18.3 ± 4.1	0.092	0.253	**0.001**
Conduit strain, %	15.2 ± 3.6	13.8 ± 4.6	13.9 ± 3.7	**0.007**	0.453	**0.005**
Booster strain, %	5.4 ± 2.4	6.0 ± 2.6	5.2 ± 2.6	0.099	**0.003**	0.374
Reservoir strain rate, 1/s	0.97 ± 0.26	1.03 ± 0.29	0.93 ± 0.22	0.229	**0.002**	0.182
Conduit strain rate, 1/s	-1.23 ± 0.35	-1.18 ± 0.44	-1.11 ± 0.34	0.218	0.312	**0.007**
Booster strain rate, 1/s	-0.72 ± 0.30	-0.74 ± 0.28	-0.66 ± 0.27	0.832	0.059	0.256
RA max. volume to RV end-diastolic volume ratio	0.42 ± 0.11	0.42 ± 0.09	0.44 ± 0.09	0.718	0.018	0.092
RA min. volume to RV end-systolic volume ratio	0.49 ± 0.17	0.50 ± 0.17	0.58 ± 0.18	0.727	**<0.001**	**<0.001**
RA stroke volume to RV stroke volume ratio	0.38 ± 0.15	0.37 ± 0.13	0.36 ± 0.11	0.614	0.429	0.158
RA max. volume to RV end-systolic volume ratio	0.99 ± 0.32	0.98 ± 0.25	1.12 ± 0.31	0.780	**<0.001**	**0.001**
RA min. volume to RV end-diastolic volume ratio	0.12 ± 0.03	0.17 ± 0.03	0.12 ± 0.03	0.455	0.028	0.239

Group characteristics presented as mean ± SD. P values represent between-group comparisons that were adjusted for differing sex and age distributions using multivariable linear regression. P_1 represents group comparisons between <32 weeks and ≥32-<37 weeks, P_2 represents group comparisons between ≥32-<37 weeks and ≥37 weeks, and P_3 represents group comparisons between <32 weeks and ≥37 weeks. P values in bold indicate statistical significance (P<0.01). LA, left atrial; LV, left ventricular; RA, right atrial; RV, right ventricular.

Figure S1. Comparisons of Reservoir, Conduit, and Booster Strain Values in Preterm-Born and Term-Born Adults.

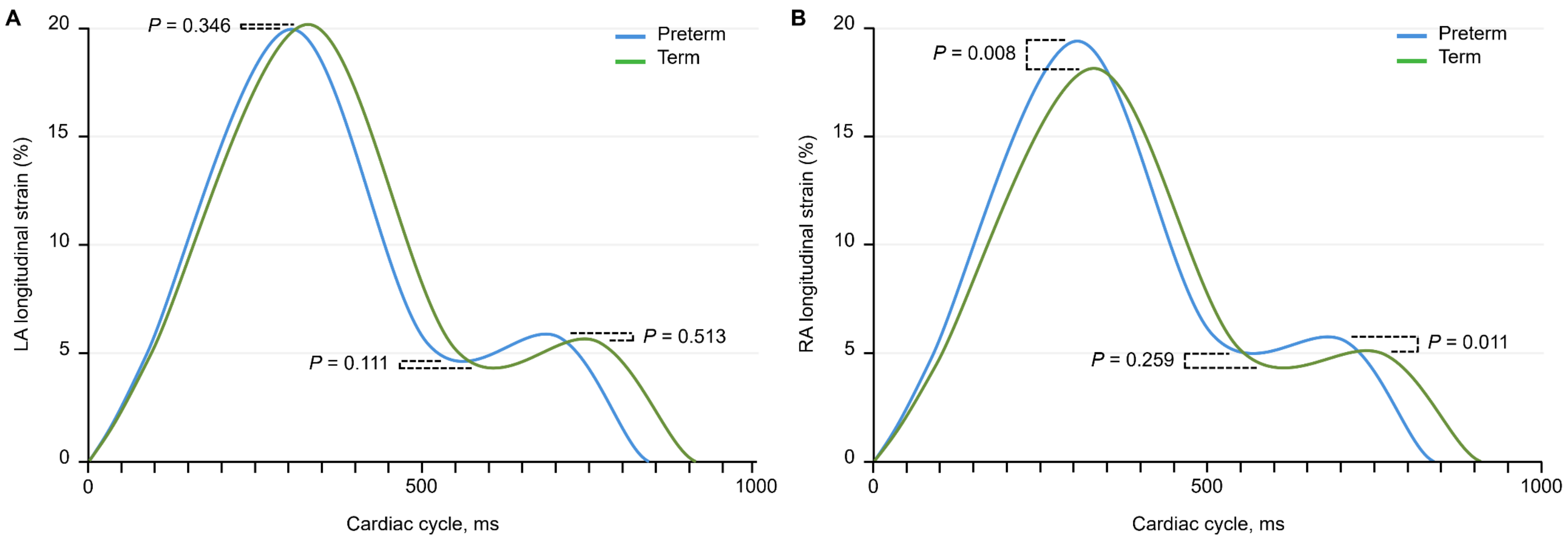

A, Visual representation of LA strain in preterm-born and term-born adults. **B,** Visual representation of RA strain in preterm-born and term-born adults. P values represent between-group comparisons for reservoir strain, conduit strain, and booster strain (from left to right, respectively) that were adjusted for differing sex and age distributions using multivariable linear regression. LA, left atrial; RA, right atrial.

Figure S2. Pooled Left and Right Atrial Strain Curves of Preterm-Born and Term-Born Adults.

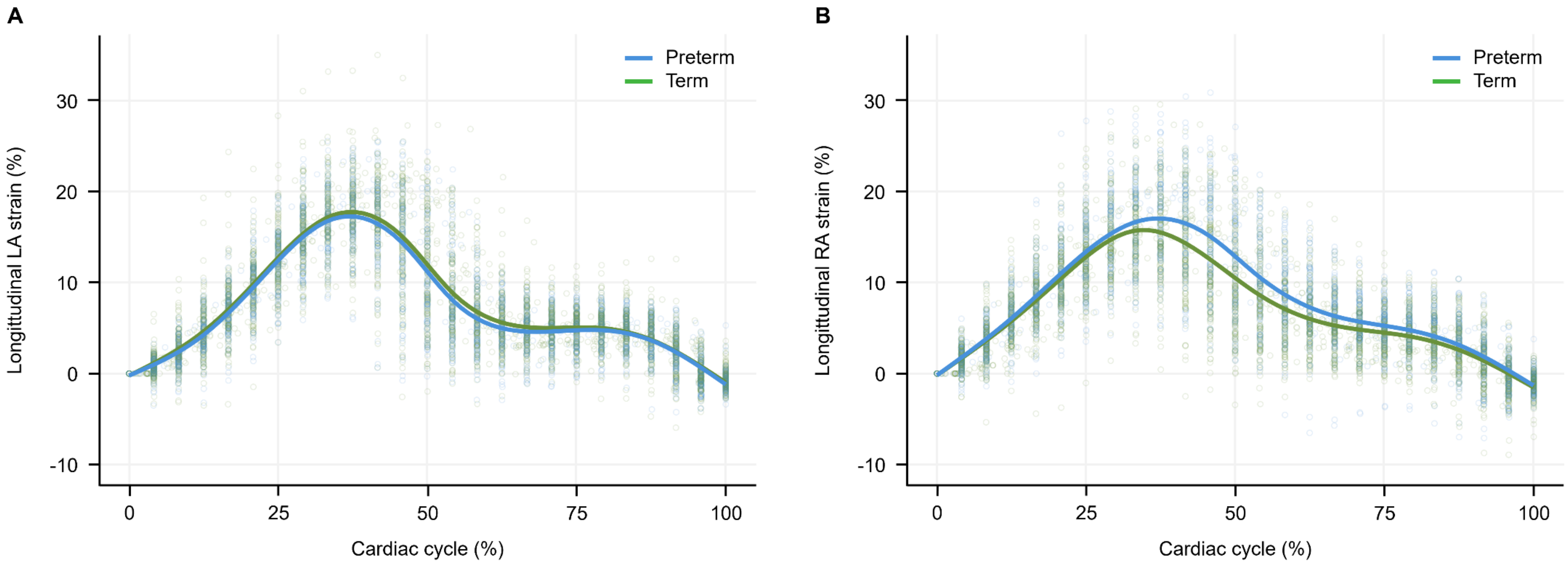

A, Pooled LA strain curves of preterm-born and term-born adults. **B,** Pooled RA strain curves in preterm-born and term-born adults. The circles indicate individual strain measurements throughout the cardiac cycle. The full lines indicate the average strain values at each point in the cardiac cycle for preterm-born and term-born adults, separately. LA, left atrial; RA, right atrial.
